# Analysis of the status quo of the Elderly’s demands of medical and elderly care combination in the underdeveloped regions of Western China and its influencing factors: a case study of Lanzhou

**DOI:** 10.1186/s12877-020-01616-6

**Published:** 2020-09-09

**Authors:** Jiancheng Wang, Yunhua Wang, Hui Cai, Juxia Zhang, Bei Pan, Guoxian Bao, Tiankang Guo

**Affiliations:** 1 Gansu Provincial Hospital, Lanzhou, 730050 People’s Republic of China; 2 Hospital Management Research Center, Lanzhou University, Lanzhou, 730000 People’s Republic of China; 3 School of Public Health, Lanzhou University, Lanzhou, 730000 People’s Republic of China; 4 School of Management, Lanzhou University, Lanzhou, 730000 People’s Republic of China

**Keywords:** The combination of medical and elderly care, Senior citizen, Demand, Analysis of influencing factors, Lanzhou

## Abstract

**Background:**

This study aims to analyse the current demand by senior citizens in Lanzhou, China for a combination of medical and elderly care services and to identify the factors influencing their needs.

**Methods:**

7500 participants aged 60 or above living in Lanzhou, China, were recruited, a unified questionnaire concerning elderly people’s demand for a service combining medical and elderly care has been adopted to survey these subjects. The status quo of the demand of the service combining medical and elderly care and its influencing factors were analysed with the single-factor Chi-square test and multi-factor binomial logistic regression method.

**Results:**

3772 of 7320 older people have the demand for the service combining medical and elderly care, accounting for 53.15% of survey respondents. Many factors are in play, including gender, marital status, degree of education, occupation before retirement, number of children, monthly income, health self-assessment status, endowment insurance type, medical insurance type, current nursing arrangements, old-age demands, self-care ability and the knowledge of combining medical and elderly care and the willingness to pay for the combination of medical and elderly care have statistical significance (*P* < 0.05) with the elderly’s needs, different ages, living styles and the prevalence of chronic diseases, have no statistical significance (*P* > 0.05) with the elderly’s care needs in Lanzhou. The number of children, type of medical insurance and willingness to pay for the combination of medical treatment and nursing care are major influencing factors among the complex factors influencing the elderly’s demand for the proposed service.

**Conclusions:**

The low knowledge rate and demand rate, the number of children, the type of medical insurance, and the willingness to pay for the medical-nursing combination service for the elderly in Lanzhou have a great impact on the elderly’s demand rate for combining medical and elderly care. Meanwhile, relevant government departments should focus more on the promotion of the endowment model of combining medical and elderly care and provide integrated medical care services by integrating multiple resources, and improving social security.

## Background

In recent years, the ageing trend has become prominent in China. In 2018, it was recorded that there were 249 million people more than 60 years old and 167 million people more than 65 years old, accounting for 17.90 and 11.90% of the total population of the country, respectively. The proportion of the population aged over 65 years old has increased year by year, and the old-age dependency ratio has also increased year by year [1]. The number of people aged more than 60 years old is expected to increase to about 255 million by 2020, accounting for 17.8% of the total population; and the old-age dependency ratio is expected to increase to around 28%; the number of elderly people of venerable age is expected to reach up to 29 million, while the elderly living alone and the empty-nested elderly are expected to reach up to 118 million [2], according to China’s ‘Thirteenth Five-Year Plan’ for the Planning of Developing the Aging Industry and Constructing the Endowment System. As the ageing trend accelerates, the number of elderly people and the disabled and semi-disabled elderly will increase in China. There were more than 40 million disabled and semi-disabled elderly people in China at the end of 2016 and 7% are cared for long term by their family. Those who need direct care also desperately require the involvement of medical services [3, 4]. Compared with developed countries in Europe and the United States, China shows a unique ageing characteristic of getting old before getting rich, at a larger scale, at a faster speed and showing a heavier dependency burden etc. What is more, China’s pension security system needs to be improved and Chinese society is facing tremendous pressure from the challenge of an ever-increasing ageing trend.

With the basic national family planning policy and economic and social transformation in play, the family supporting function has been weakening, while elderly people’s demands for professional nursing institutions and community services have been steadily on the increase. In particular, people aged over 80 years old with a high morbidity rate of chronic disease desperately need systematic, comprehensive, convenient and low-cost medical services. Moreover, both medical and elderly care, as a matter of record, are indispensable for the disabled and semi-disabled elderly [5]. The elderly’s medical needs cannot currently be satisfied either in most of the old-age nursing institutions that provide low-level medical services with only a few qualified nursing staff and limited beds or in medical institutions which cannot provide long-term hospitalisation services for the elderly due to their limited resources. Besides, care resources at the community level cannot fully cover the medical and nursing demands of the disabled and non-disabled elderly suffering from diseases.

The ageing of the population has exacerbated the shortage of resources for medical services and elderly nursing, which has put forward a request for improving the allocation and utilisation of social resources. The traditional elderly nursing model cannot satisfy the all-round care needs of the elderly. It is therefore imperative to implement a new model of health care for the elderly. Nevertheless, medical treatment and eldercare resources are inadequately supplied and mutually independent, which, therefore, cannot meet the needs among the elderly nowadays. Therefore, it is of great necessity to provide them with a ‘medical–nursing combination’ service that organically combines medical and elderly care.

Since there is no standard definition of a ‘medical–nursing combination’ in China, it is defined differently by a wide range of scholars. Guo et al. believe that the ‘medical–nursing combination’, denotes a process that gradually forms a cooperative service model integrating medical treatment, recovery and nursing from service providers (including hospitals, elderly nursing institutions and communities) providing a medical and nursing service conforming to elderly nursing to the elderly in demand according to different health needs at different stages of diseases [6]. Liu et al. defined the combination of medical care and nursing as satisfying the needs of health problems at different levels for the elderly at different stages in the care process, through integrating medical resources and pension resources to optimise the allocation of medical and nursing resources [7]. Liu et al. consider that elderly people can achieve the purpose of obtaining medical treatment while suffering from diseases, and enjoying care while not suffering from diseases under the new elderly nursing model combining medical and elderly care [8]. To Huang et al., the medical-nursing combination possesses the same concept as ‘long-nursing’ overseas, which focuses on meeting the basic living needs of the elderly, as well as physical and psychological care; moreover, medical treatment should be highlighted, while the enhancement of daily living skills, the adaptation of social environment and the realisation of self-worth are also important [9].

To solve the medical problem of the ageing population, the concept of a medical-nursing combination was first proposed in ‘Several Opinions on Accelerating the Development of the Elderly Nursing Service Industry’ issued by the State Council in September 2013. It pointed out the need to provide multi-level elderly nursing services, actively respond to the ageing population and accelerates the development of the elderly nursing service industry through actively driving the combination of medical and elderly care services. ‘Guiding Opinions on Promoting the Combination of Medical Treatment and Elderly nursing Services’ issued by the State Council in November 2015 indicated two tasks for promoting the combination, firstly, encouraging elderly nursing institutions to conduct various forms of agreement and cooperation with other medical and health institutions and establish a sound cooperation mechanism; secondly, promoting the extension of medical and health services to communities and families. The ‘Thirteenth Five-Year Plan’ for the Planning of Developing the Aging Industry and Constructing the Endowment System issued by the State Council in March 2017, focused on assigning nine tasks including the active promotion of the medical–nursing combination service and improving the allocation and utilisation of social resources. As of 2017, China has set up 90 national-level pilot cities for combining medical and elderly care [10].

On that basis, the ‘medical and elderly care combination’ is a new elderly nursing model that provides the elderly with services such as uninterrupted daily care, mental consolation, disease diagnosis and treatment, health guidance, recovery from serious illnesses and hospice care through effectively integrating medical and elderly care resources to satisfy the varied health care needs of the elderly at multiple levels.

Currently, four medical and elderly care models can be found in China [11]. The first model is ‘nursing in hospital’, that is, a geriatric department is set in some large hospital with conditions to provide medical treatment, nursing, care for the elderly, rehabilitation, health education, hospice care and similar services; or some low-level primary hospitals with spare resources are transformed into nursing institutions for medical rehabilitation, convalescence and elderly nursing, to achieve the goal of integrating medical and elderly care. The second model is ‘constructing a hospital in nursing institutions’, and providing professional medical and nursing teams according to the standards of national hospitals in large-scale elderly nursing institutions or welfare homes. Meanwhile, basic medical departments such as a comprehensive medical-surgical department, rehabilitation department and pharmacy are set up to form a new elderly nursing institution, integrating elderly nursing with healthcare functions. The third model is the union of medical and elderly care, namely, a cooperation mechanism is established between medical institutions and elderly nursing institutions. In this way, medical institutions provide medical care training to nursing staff in elderly nursing institutions, and regularly conduct basic diagnosis and treatment services such as detection of common diseases, the management of chronic and geriatric diseases, as well as health education. Meanwhile, the hospital also offers a green channel to provide a timely medical referral service for the elderly in need, and conduct subsequent recovery treatment in the eldercare institutions after the individual’s condition is controlled. By doing so, a two-way continuous care model is generated. The fourth model is ‘home nursing’, which is, in essence, a family doctor model. A service team provides outpatient services and life nursing services for the elderly. It is a model that is primarily designed for the elderly in good health, allowing them to enjoy their old age in peace with familiar surroundings.

As developed western countries entered the ageing society paradigm earlier than China, they have developed a new elderly nursing model called ‘long-term nursing’ that is consistent with the medical-nursing model in concept, connotation, service purpose, content and objects. The United Kingdom, the United States and Japan are the most typical countries that have developed their unique representative research results concerning elderly nursing.

The elderly nursing model in the United Kingdom is dominated by community and home care. The main service providers are composed of managers, professional staff and caregivers, who provide four major services including life care, material support, psychological support and overall care. Specifically, life care is mainly to provide home-care services and short-term care services for the self-care or semi-self-care elderly; material support includes the government upgrading the infrastructure of the elderly’s living place and providing tax subsidies or preferences to taxpayers more than 65 years old; psychological support is where service staff visit the elderly for health inspection, publicising health care knowledge, making rehabilitation and treatment suggestions and providing psychological counselling; overall care is delivered in community activity centres funded by the government or the society and is designed to inject fun into elderly people’s lives, and some low-intensity jobs are provided to increase the elderly’s income and maintain their mental health [12].

The elderly nursing model combining medical and elderly care in the US is dominated by a programme of all-inclusive care for the elderly (PACE) that is set up for the disabled, the semi-disabled and over-55-year-old low-income groups requiring long-term medical care. Covering medical services, rehabilitation services and social support services, the purpose of PACE is to assist the elderly and the debilitated to live as long as possible in the community or family, improving the living quality of the elderly with weak self-care ability and maximally protecting the dignity of the elderly [13]. The elderly nursing combining medical and elderly care model in Japan is dominated by the following models. First, the daycare centre; this model mainly provides rehabilitation and life care services for the elderly of more than 65 years old who are unattended at home in the daytime and need rehabilitation training. Second, the nursing centre; this is supported by a service consisting of nurses, caregivers and welfare workers to provide daily services for the disabled elderly living in the centre. Third, the elderly welfare centre is targeted at the elderly in the community; service staff, mainly health care therapists, provide services including health examination, health education, health care services and family guidance. Fourth is the apartment for the elderly, which is mainly designed for the healthy elderly who can take care of themselves. It provides basic medical services and daily care services. An all-round legal system is a major reason why the elderly nursing combining medical and elderly care has been well developed in Japan [14].

Research on the ‘combination of medical and elderly care’ have been identified abroad with proven systems moving from policies to actual services, which can provide a reference and basis for researching and implementing the combination of medical and elderly care in China. Researching the ‘combination of medical and elderly care’ service model is still in its initial stage of development in China. Related research studies in the domestic literature focus on introducing and analysing foreign elder care cases based on the ‘combination of medical and elderly care’, which propose the status quo of the development of the ‘combination of medical and elderly care’ model before making suggestions or conducting a case study in the pilot region of combining medical and elderly care in China. Nevertheless, few studies cover the service requirement and influencing. Instead, most of the research discusses elderly people’s basic situation, health status, social support and income status, etc. Generally speaking, the better the health condition of the elderly, the higher the self-care level, the lower the income and the lower the social support, the less demand for a service combining medical and elderly care [11, 15]. Li et al. found that the number of children, health status, children’s support and willingness to pay have significant impacts on the demand for services combining medical and elderly care after investigating more than 420 elderly people aged more than 60 years in four major urban areas of Chongqing [15]. Hu et al. discovered that degree of education, ideal method of elderly nursing and willingness to pay are significant factors affecting the elderly’s demand for the service combining medical and elderly care in urban areas after surveying the elderly in Yinchuan [16]. According to Zhou et al.’s research, age, degree of education, number of children and occupation type before retirement are main factors affecting the elderly’s demand for the new service [17]. Through investigation, Wang et al. believe that the elderly in Changchun have a high willingness to participate in a combined service. Gender, age, education and occupation type are major factors affecting their choices [18].

The basis of medical insurance in China is comprised of a basic medical insurance system for urban workers, a basic medical insurance system for urban residents and a new rural cooperative medical insurance [19, 20]. A unified basic medical insurance system for urban and rural residents should be gradually established nationwide according to the Opinions on Integrating the Basic Medical Insurance System for Urban and Rural Residents issued by the State Council in 2016. The number of people insured with basic medical insurance in China has exceeded 1.35 billion with a participation rate of over 95% by the end of 2017, basically realising a full coverage from ‘insurance for few’ to ‘insurance for all’ [21].

The basic medical insurance system for urban employees is raised jointly by social medical unified planning and individual account, forming a social medical unified planning fund and individual medical account fund. The individual account is not set in the basic medical insurance system for urban and rural residents. In other words, only the social medical unified planning fund is established to raise funds through quota. The premiums consist of individual residents’ contributions and financial subsidies.

Most scholars in our country believe that the demand for the service of combining medical and elderly care is affected by the design and implementation of medical insurance systems and the elderly’s capacity to pay under the current medical insurance system. In terms of system design, there is a lack of long-term care insurance specifically for elderly nursing, and the elderly nursing service combining medical and elderly care is not involved in the designated medical insurance units. Medical insurance in China focuses on economic compensation for the loss caused by a given disease and lacks compensation for preventive health care, rehabilitation, long-term care and similar services needed by the elderly, whereas basic pensions are mainly used for daily care of the elderly [22]. On the aspect of system implementation, the reimbursement practice of medical insurance in China is characterised by ‘designated medical care with three medical directories’. Since setting up medical institutions in nursing institutions is not included in the designated medical organisation, additional medical services in the nursing institution cannot be paid through medical insurance. In this case, the elderly living in the nursing institution have to visit hospitals for treatment, reducing access to medical services [23]. Besides, a plurality of issues such as the admission of nursing institutions, the verification of medical qualifications, medical insurance designated hospitals and review and distribution of charges can be found in the nursing institution involved in combining medical and elderly care [24]. Regarding the elderly’s capacity to pay, the medical insurance only covers medical expenses and examination costs incurred during the medical process. With the lack of a long-term care insurance system, rehabilitation medical programmes, life care programmes and auxiliary equipment programmes are fully incurred by the elderly. However, elderly patients who are economically disadvantaged, especially the disabled and the semi-disabled, the elderly suffering from diseases and more than 80 years old have a limited capacity to pay for the long-term care cost [25].

The elderly are the main service object of the ‘medical–nursing combination’ model, whose demand willingness plays a decisive role in the development of the ‘combination of medical and elderly care. Therefore, it is essential to proceed from the elderly’s demand for specific services before conducting an in-depth exploration of the new model.

**Fig. 1:**
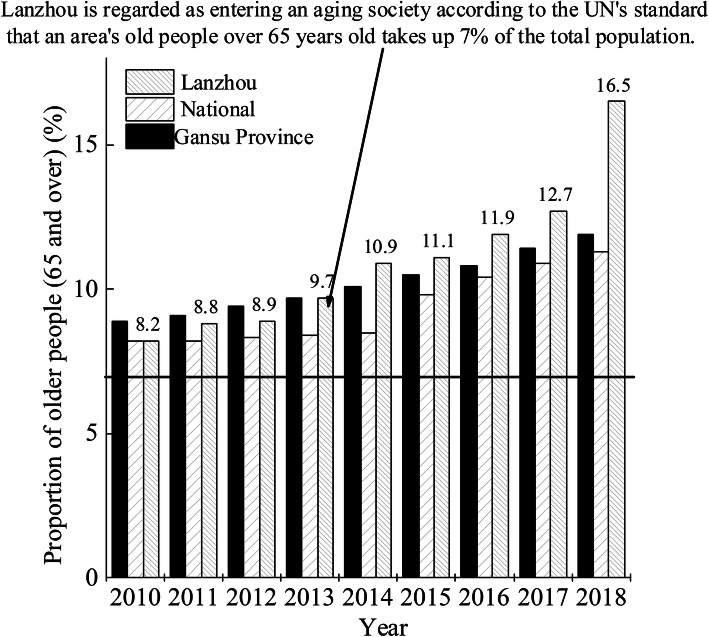
Population aging trend in Lanzhou city from 2010 to 2018

As of the end of 2018, the number of elderly people aged 65 years old or above in Lanzhou has reached 498,800, accounting for 16.50% of the total population [26]. Moreover, the proportion of the population aged 65 years old or above has been higher than the average level of the whole country and Gansu during the same period (See Fig. 1. Data source, National data from 2010 to 2018 were from the China Statistical Yearbook [26] and data of Gansu from 2010 to 2018 were from the Gansu Statistical Yearbook [27], data of Lanzhou from 2010 to 2018 were obtained from the Lanzhou Yearbook [28]). As can also be seen from Fig. 1, the degree of the ageing phenomenon has become more serious in Lanzhou from 2010 to 2018, in which the ageing rate was 8.20% in 2010 and jumped to 16.50% in 2018, indicating that the growth of the ageing population has been accelerating by 8.3%. By comparison, the national ageing rate was 11.9% in 2018, which clearly shows that the ageing rate in Lanzhou had accelerated. What is worse, the ageing problem in Lanzhou would be crucial, as the degree of ageing population could become serious over time. The accelerating population ageing in Lanzhou has put tremendous pressure on elderly nursing. Furthermore, elderly nursing involves a variety of requirements such as medical rehabilitation and spiritual happiness with the social progress, rather than merely basic daily care. It can be seen that a tremendous requirement has been proposed to develop multi-integrated nursing services combining nursing and medical treatment based on the huge elderly group and the serious ageing status quo in Lanzhou.

Incomplete statistics show that there are 27 nursing institutions in Lanzhou as of now, including seven institutions run publicly and 16 run privately, and four institutions combining medical and elderly care, providing a total of 6107 beds. Specifically, 18 hospitals have set up geriatric services and geriatric beds, providing a total of 500 beds, accounting for 69% of the total number of hospitals; 26 hospitals above the county level have set up green medical treatment channels for the elderly; and 19 nursing institutions can provide medical services, accounting for 70.4% of the total nursing institutions; the contracted service rate of the home-based elderly aged more than 65 years old in Lanzhou reached 73% [29, 30]. As a national pilot city for combining medical and elderly care, Lanzhou has made some progress in the process of developing a service combining medical and elderly care. However, the follow-up work remains cumbersome since the policy obstacles in the combination of medical and elderly care should be overcome. What is more, concrete service content and links should be improved, such as constrained nursing conditions in medical institutions, missing service function of nursing institutions, high cost, constrained reimbursement of medical expenses, pessimistic cognitive status of the concept of combination of medical and elderly care and the institution management system requiring enhancement [30].

Scholars tend to be more willing to concentrate on the process and obstacles of combining medical and elderly care at the macro level for such a new type of elderly nursing. However, few studies analysing the elderly’s needs for a combined medical and elderly care services can be found.

The social and economic foundation of the undeveloped region of western China is relatively weak with a low level of social security and welfare. In particular, the elderly long-term care system in remote rural areas is in its infancy. Lack of a well-defined medical- nursing mechanism seriously affects the well-being and happiness of residents in the area. As the driving strategy of combining medical and elderly care has been vigorously promoted at the national level, theoretical introduction and countermeasure are essential to regional strategic layout. A questionnaire survey concerning the need for an elderly nursing service combining medical and elderly care was conducted on residents in Lanzhou. On this basis, the specific needs of residents for elderly nursing services combining medical and elderly care were analysed. By sorting out factors affecting the demand for elderly nursing service combining medical and elderly care, policy proposals were made accordingly. The case study of Lanzhou was taken as an example to provide a referring significance of developing a combination of medical and elderly care in the undeveloped region of western China, to raise the health care levels of residents in the undeveloped region of western China, satisfy their medical and nursing requirements and improve their nursing services.

## Methods

### Participants

**Fig. 2:**
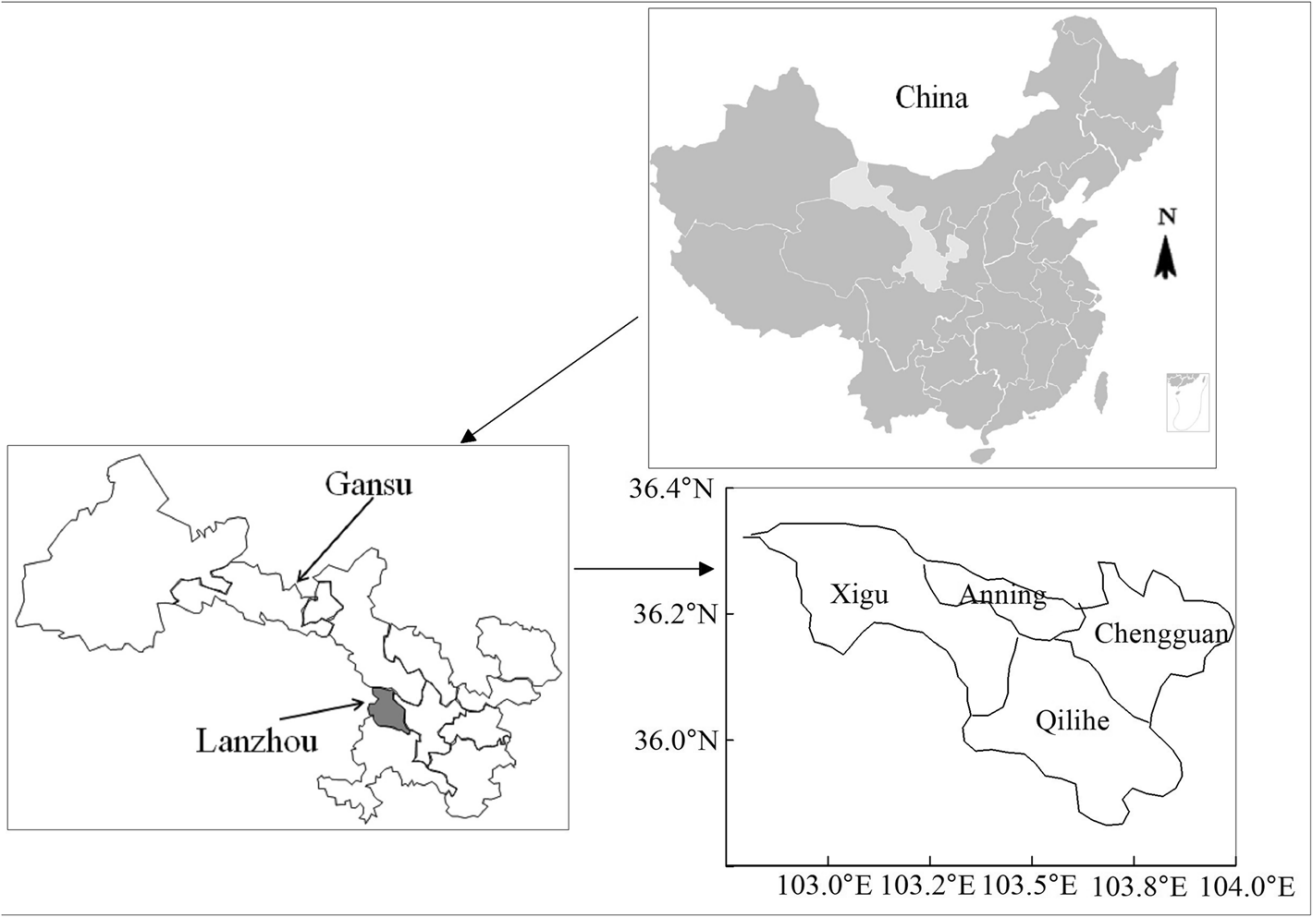
The geographical location of the study area in China. (The maps were downloaded from the following website: http://wenku.baidu.com/view/fa91624477eeaeaad1f34693daef5ef7bb0d1252. The above maps are available free of charge, and we redrew the map according to the requirements of the article)

A questionnaire survey was conducted in four districts of Lanzhou (including Chengguan District, Qilihe District, Anning District and Xigu District; the location map of the study area is shown in Fig. 2) through stratified random sampling. Elderly residents, aged 60 years old or above were selected as survey subjects. A total of 7500 elderly people were surveyed with the distribution of the questionnaire concerning the eldercare service provision combining medical and elderly care.

Selection criteria: ①age ≥ 60; ②length of residence ≥ 6 months; ③elderly respondents without known significant cognitive impairment, severe illness and terminal illness and elderly people without visual and hearing impairment caused by various reasons; ④informed consent had to be sought and secured. 7500 questionnaires in total were administrated this time, and 7320 valid ones were collected.

### Design and procedure

Questionnaire concerning elderly people’s needs for combined medical and elderly care: Questionnaires covering personal characteristics, health status, economic status and elderly people’s understanding of a combined medical–nursing service, have been frequently adopted by scholars at home and abroad for investigating the need for an elderly nursing service combining medical and elderly care. To better compare with the results of related literature [11–18, 31–42], and perform an in-depth analysis of factors affecting the need for combining medical and elderly care, a questionnaire related to the eldercare needs of combining medical and elderly care has been designed by epidemiological and statistical experts and jointly compiled by the research team and elderly nursing management experts based on a full reference to relevant literature [11–18, 31–42] and national health policies. The in-house designed ‘Survey on the elderly’s needs of combination of medical and elderly care in Lanzhou’ was used as a survey instrument. The questionnaire was designed following the literature [11–18, 31–42], the China Health and Pension Tracking Survey and the Fifth National Health Service Survey-Family Health Questionnaire, as shown in Table 1.

**Table 1:** Summary of questionnaires regarding Lanzhou senior citizens’ demand for medical and elderly care combination and influence factors

Questionnaire	Number of Items	Investigation Index	Reference or Citing Source
General demography	6	Gender, age, education background, marital status, pre-retirement occupation, number of offspring	[12–19, 32–43], Tracking Survey of Health and Elderly Care in China
Health condition	3	Self-assessed health condition, incidence of chronic disease, activity of daily living	[12–19, 32–43], Tracking Survey of Health and Elderly Care in China, the Fifth National Health Service Survey-Family Health Inquiry Questionnaire
Medical endowment insurance	5	Medical insurance, endowment insurance, monthly income, living status, current elderly care method	[12–19, 32–43], Tracking Survey of Health and Elderly Care in China, the Fifth National Health Service Survey-Family Health Inquiry Questionnaire
Understanding of and demand for the combination with medical care	4	Knowledge of the combination with medical care, demand for the combination with medical care, the current way of old-age care, willingness to pay in combination with medical care	[12–18, 32–43]

Before the questionnaire was officially distributed, 100 retired residents were selected for pre-survey through convenience sampling. And a formal questionnaire was finalised after the questionnaire was revised according to the pre-survey results. The questionnaire is composed of general demographics, health status, medical endowment insurance and the knowledge of and expressed a requirement for combining medical and elderly care. A detailed questionnaire is provided by us as Supplemental Materials. See the Supplemental Materials.

### Quality control method

The survey was conducted face to face by trained investigators familiar with working with elderly respondents. Investigators explained related concepts in the questionnaire during the survey considering that the elderly were not understanding the combination of medical and elderly care. Questionnaires were collected and checked by a specially-assigned person who was responsible for removing the invalid and the incomplete questionnaires. Questionnaires that qualified based on the investigators’ reviews were retrieved in time.

### Statistical analyses

Software Epidata3.1 was used to log and proofread data in duplicate. Statistical software SPSS 18.0 was used for statistical treatment. The enumeration data used *χ*^2^ test, and the influencing factors were analysed with a binomial logistic regression. Statistical significance in the differences would be confirmed in case of *P* < 0.05.

## Results

### Sociodemographic characteristics of senior citizens in Lanzhou

Table 2 presents the basic characteristics of the elders enrolled in the current study. Retired elderly people aged 60 years old or above were surveyed. Among them, the number of males and females are 3059, and 4261, accounting for 41.79, and 58.21%, respectively; and the elderly aged between 60 and 70 years old are 77.62%, aged between 71 and 80 years old are 21.64%, and aged more than 80 years old are 0.74%; also, there are 404 (5.52%) elderly people with elementary education or below, followed by 1437 (19.63%) with junior high school and 2560 (34.97%) with senior high school or technical secondary education, 2380 (32.51%) with junior college education and 539 (7.37%) with undergraduate or above. Moreover, 50% of the surveyed elderly who had worked in enterprises and public institutions before retirement enjoy stable retirement pay; 14.51% of the surveyed elderly have one child, 29.59% have two children and 55.90% have three children. In the survey, 3388 elderly people considered that they were in good health, accounting for 46.28%, while 44.64% considered their health to be not good enough and 664 elderly people (about 9.07%) considered they have poor health. The prevalence of chronic diseases among the elderly involved in the survey is 50.46%; 5231 elderly people can take care of themselves, accounting for 71.46%; while 1890 and 199 elderly people need help from others and cannot take care of themselves, respectively, accounting for 25.82 and 2.72%, respectively. 3930 elderly people have basic medical insurance for urban and rural residents, accounting for 53.69%; but only 590 elderly people have commercial medical insurance, accounting for 8.06%; 983 elderly people have no medical insurance, accounting for 13.43%; up to 3791 elderly people have endowment insurance for urban employees, accounting for 51.79%; the smallest proportion of elderly people, only 111 have commercial endowment insurance, accounting for 1.52%. There are 706 elderly people who do not have any endowment insurance, accounting for 9.64%; 2378 elderly people have a monthly income ranging from 2000 to 4000 yuan, accounting for 32.49%, followed by 2192 people with a monthly income ranging from 4000 to 6000 yuan and 1412 people with a monthly income larger than 6000 yuan. There are 5902 people choosing to live at home, accounting for 88.32%; people living alone account for 19.37% and 5902 (accounting for 80.63%) people live in other ways. More details are presented in Table 2.

**Table 2:** Comparisons of old-age demand on "medical-nursing combination" with different social demographic characteristics of the elderly

Independent variable	Number	%	Demand rate	Chi-square	*P*
Gender					
Male	3059	41.79	1749(57.18)	6.67	0.000
Female	4261	58.21	2023(47.48)		
Age (years)					
60-70	5682	77.62	2814(49.52)	3.635	0.162
71-80	1584	21.64	923(58.27)		
>80	84	0.74	35(41.67)		
Marital status					
Married	5812	79.40	2913(50.12)	9.46	0.000
Unmarried	81	1.11	10(12.35)		
Divorced or widowed	1427	19.49	84(5.89)		
Education					
Elementary school and below	404	5.52	183(45.30)	12.54	0.000
Junior high school	1437	19.63	757(52.68)		
Senior high school or technical secondary school	2560	34.97	1217(47.54)		
Junior college	2380	32.51	1265(53.15)		
Bachelor degree or above	539	7.37	350(64.94)		
Pre-retirement occupation					
State functionary	472	6.45	294(62.29)	43.50	0.000
Public institution personnel	1437	19.63	946(65.83)		
Enterprise staffs	2403	32.83	1123(46.73)		
Other	3008	41.09	1409(46.84)		
Number of children					
0	0	0.00		33.20	0.000
1	1062	14.51	799(75.24)		
2	2166	29.59	1237(57.11)		
≥3	4092	55.90	1736(42.42)		
Self-rated health					
Good	3388	46.28	1771(41.57)		
Fair	3268	44.64	1725(52.27)	31.027	0.000
Poor	664	9.07	276(52.78)		
Have chronic diseases					
Yes	3694	50.46	1955(52.92)		
No	3626	49.54	1817(50.11)	1.395	0.901
Self-care ability					
Have self-care ability	5231	71.46	2041(39.02)		
Need help from others	1890	25.82	1572(83.17)	43.38	0.000
No self-care ability	199	2.72	159(79.90)		
Type of medical insurance					
Medical insurance for urban workers	1817	24.82	958(52.72)		
Medical insurance for urban and rural residents	3930	53.69	2065(52.54)	37.18	0.000
Commercial insurance	590	8.06	477(80.85)		
Not have	983	13.43	272(27.67)		
Type of endowment insurance					
Endowment insurance for urban workers	3791	51.79	1992(52.55)		
Endowment insurance for urban and rural residents	2378	32.49	1142(48.02)		
Enterprise annuity	334	4.56	223(66.77)	29.87	0.000
Commercial insurance	111	1.52	50(45.05)		
Not have	706	9.64	335(47.45)		
Income per month (RMB)					
< 2000	1338	18.28	593(44.32)		
2000-4000	2378	32.49	1207(50.76)	17.85	0.000
4000-6000	2192	29.95	1142(52.10)		
> 6000	1412	19.29	830(58.78)		
Living arrangement					
Not alone	5902	80.63	3079(52.17)	4.708	0.079
Alone	1418	19.37	693(48.87)		
The current way of old-age care					
Family endowment	6465	88.32	3177(49.14)		
Community endowment	632	8.63	446(70.57)	38.20	0.000
Institution endowment	223	3.05	149(66.82)		
Other	0	0.00			
Knowledge of the combination with medical care					
Never heard	4570	62.43	2093(45.80)		
Heard but not understood	2155	29.44	1304(60.51)	56.41	0.000
Have gained some understanding	557	7.61	369(66.25)		
Know well	38	0.52	6(15.79)		
Demand of the elderly’ care					
Medical care	1806	24.67	1101(60.96)		
Assisted living	3733	51	1575(42.19)	57.81	0.000
Mental care	220	3.01	122(55.45)		
Leisure and entertainment	1561	21.33	974(62.40)		
Willingness to pay in combination with medical care per month (RMB)					
< 1000	1040	14.21	380(36.54)		
1000-2000	1561	21.33	674(43.18)	69.64	0.000
2000-3000	2192	29.95	1088(49.64)		
> 3000	2527	34.52	1630(64.50)		

### Understanding of and demand for the combination of medical and elderly care by senior citizens in Lanzhou

Table 2 also displays an understanding of and demand for the combination of medical and elderly care for different sociodemographic characteristics of the elderly. Among the 7320 elderly people surveyed, 62.43% have never heard of the combined service, 29. 44% have heard of it but do not understand it; only 7.61% have a basic understanding of the model and only 38 people (accounting for 0.52%) have understood this model well. Meanwhile, 3772 out of the 7320 elderly people surveyed have a demand for the service combining medical and elderly care, accounting for 51.53%. Also, 41.75% of those who have a demand for a service combining medical and elderly care most want to obtain life care in the combined service, and nearly half of the elderly most want a medical care service integrating prevention, health care, medical treatment, with rehabilitation.

### Single factor analysis of the influencing factors on the demand by senior citizens in Lanzhou for the combination of medical and elderly care

Through analysis, different genders, marital status, degree of education, occupation before retirement, number of children, monthly income, health self-assessment status, endowment insurance type, medical insurance type, current way of elderly nursing, old-age demands, self-care ability and knowledge about combining medical treatment and health care, it is found that the willingness to pay for the combination of medical and elderly care have statistical significance (*P* < 0.05) with the elderly’s needs for a combination of medical and elderly care in Lanzhou, whereas different ages, living styles and the prevalence rate of chronic diseases display no statistical significance (*P* > 0.05) with the elderly’s needs for a service combination in Lanzhou. More details are presented in Table 2.

### Logistic regression analysis of the influencing factors on the demand by senior citizens in Lanzhou for the combination of medical and elderly care

Factors (including gender, marital status, degree of education, occupation before retirement, number of children, monthly income, health self-assessment status, endowment insurance type, medical insurance type, current model of elderly nursing, old-age demands, self-care ability and knowledge about combining medical treatment and health care and the willingness to pay for the combination of medical and elderly care) with statistical significance in the single-factor analysis were regarded as independent variables Xi; the elderly’s demand for the service combining medical and elderly care was taken as a dependent variable Y (Y = 1 indicates a demand for the service combining medical and elderly care, and Y = 0 indicates no demand for the service combining medical and elderly care) for conducting binomial logistic regression analysis. The relationship between the elderly’s demand for the service and various variables were obtained, as shown in Table 3.

**Table 3:** Variable definition for related factors and demand of the medical-nursing combined service

Variable Categories	Variable Name	Code
Gender	X1	Male =1,Female = 2
Marital status	X2	Married = 1, Unmarried = 2, Divorced or widowed = 3
Education	X3	Elementary school and below = 1, Junior high school = 2, Senior high school or technical secondary school = 3, Junior college = 4, Bachelor degree or above = 5
Pre-retirement occupation	X4	State functionary = 1, Public institution personnel = 2, Enterprise staffs = 3, Other
Number of children	X5	0 = 1, 1 = 2, 2 = 3, ≥ 3 = 4
Income per month(RMB)	X6	<2000 = 1, 2000 ~ 4000 = 2, 4000 ~ 6000 = 3, >6000 = 4
Self-rated health	X7	Good = 1, Fair = 2, Poor = 3
Type of endowment insurance	X8	Endowment insurance for urban workers = 1, Endowment insurance for urban and rural residents = 2, Enterprise annuity = 3, Commercial insurance = 4, Not have = 5
Type of medical insurance	X9	Medical insurance for urban workers = 1, Medical insurance for urban and rural residents = 2,Commercial insurance = 3, Not have = 4
The current way of old-age care	X10	Family endowment = 1, Community endowment = 2,Institution endowment = 3, Other = 4
Demand of the elderly	X11	Medical care = 1, Assisted living = 2, Mental care = 3, Leisure and entertainment = 4
Self-care ability	X12	Have self-care ability = 1, Need help from others = 2, No self-care ability = 3
Knowledge of the combination with medical care	X13	Never heard = 1, Heard but not understood = 2, Have gained some understanding = 3, Know well = 4
Willingness to pay in combination with medical care per month(RMB)	X15	<1000 = 1, 1000 ~ 2000 = 2, 2000 ~ 3000 = 3, >3000 = 4
Demand in combination of medical care	Y	Yes = 1, No = 2

Influencing factors were screened using the stepwise regression method. The inclusion criterion was α = 0.05 and the removal criterion was α = 0.1. The results of logistic regression on the combined service need are shown in Table 4. According to regression results, major influencing factors are comprised of the number of children, health self-rating, type of medical insurance, current model of elderly nursing, elderly nursing need, self-care ability of daily living, prior or acquired knowledge of the proposed service model and willingness to pay for the combination of medical and elderly care (*P* < 0.05, see Table 4).

**Table 4:** Logistic regression analysis including variables associated with medical-nursing combined service demand for the elderly

Independent variable	*β*	*SE*	*P*	*OR*	95%CI for *OR*	
					Lower	Upper
Gender	0.62	0.15	0.00	1.25	0.95	2.32
Marital status	0.27	0.14	0.09	0.93	0.59	1.47
Education	0.34	0.08	0.15	1.17	0.92	2.49
Pre-retirement occupation	0.14	0.09	0.00	1.15	0.90	1.26
Number of children	0.50	0.13	0.00	1.39	1.03	2.15
Income per month (RMB)	0.25	0.17	0.19	1.21	0.87	1.91
Self-rated health	-0.46	0.12	0.00	0.67	0.52	0.83
Type of endowment insurance	-0.12	0.11	0.43	0.93	0.67	1.23
Type of medical insurance	0.59	0.22	0.00	1.65	1.23	2.45
The current way of old-age care	-0.43	0.18	0.01	0.61	0.42	0.91
Demand of the elderly	-0.63	0.17	0.00	0.53	0.37	0.72
Self-care ability	-1.23	0.28	0.00	0.34	0.12	0.65
Knowledge of the combination with medical care	-0.59	0.32	0.00	0.71	0.39	0.83
Willingness to pay in combination with medical care per month (RMB)	0.90	0.34	0.00	1.94	1.17	2.64

## Discussion

### Comparison of the understanding of and demand by senior citizens for the combination of medical and elderly care services

Surveyed data of this study show that only 8.13% of the 7320 elderly people aged more than 60 years old in Lanzhou knew about and understood the service combining medical and elderly care. Among senior residents in Datong 25.0% were aware of the model of combining medical and elderly care, while only 6.0% understood well the model of combined medical and elderly care [11]. As can be observed from the study conducted by Hu et al. in Yinchuan, only 7.6% of the residents had a basic knowledge about the elderly nursing model of combining medical and elderly care and only 0.5% understood the combination model well [16]. Studies conducted by Zhou el al. in Urumchi show that 13.67% of the residents had heard of or at least knew about the service [17]. According to research conducted by Wang et al. in Changchun, 2.54 and 6.21% of the elderly people in the community well understood and had a basic knowledge of the elderly nursing model combining medical and elderly care, respectively [18]. The study conducted by Liu et al. in Karamay found that only 11.30% of the local residents knew and understood the proposed combined model [37]; and the study conducted by Wang et al. in Beijing shows that 12% of the elderly residents understood the model, while only 4.0% of those understood and actually used the combined model [38]. The results shown in this study are basically consistent with the above findings, indicating a low knowledge rate of the combined service model.

The combination of medical and elderly care is still at the exploratory stage in China and few investigations could be found on the demand rate for some combination of medical and elderly care, since most of the related research is dominated by theories, problems and countermeasures. This survey shows that the demand rate for the service combining service provision for the elderly in Lanzhou is 51.53%. The elderly people’s demands for this service were also reported in some domestic studies, for example, the demand for the elderly nursing service combining medical and elderly care for the elderly in cities and towns in Datong makes up 50.5% [11]; the demand for the service for the elderly in Chongqing is 53.00% [15]; the demand for the service for the elderly in Yinchuan accounts for 50.80% [16]; 56.21% of the elderly in Changchun demand the combined service [18]; the demand for the service for the elderly in downtown Zhanjiang is 54.60% [31]; 61.10% of the empty-nested elderly in Quanzhou communities need the service [32]; the demand for the service for the elderly in Karamay is 53.01% [37]; the demand for the service for retired residents in Tianjin is 61.9% [39]; a strong demand for the elderly nursing service combining medical and elderly care for elderly patients can be witnessed in Weifang, accounting for 97.40% [40] and 43.40% of the elderly in Qiqihaer have a demand for the combined service [41]. Compared with the above studies, a lower demand for the elderly nursing service combining medical and elderly care can be observed in Lanzhou.

It is generally recognised that the knowledge rate and the service demand for combining medical and elderly care are directly proportional, namely, the higher the knowledge rate, the higher the demand for the service combining medical and elderly care [31, 42]. For example, the research conducted by Wu et al. in Zhanjiang proves that elderly people’s knowledge rate of the service combining medical and elderly care in downtown Zhanjiang is 59.40%, and the demand rate for the service combining medical and elderly care is 54.60% [31]. Nevertheless, some studies also show that the knowledge rate of the service is not a factor influencing the demand for the service. For example, the studies conducted by Hu et al. in Yinchuan and by Liu et al. in Karamay show that the knowledge rates of the service are 7.60 and 16.11%, respectively, and their corresponding demand rates for the service are 50.80 and 53.01%, respectively [16, 37]. These figures are inconsistent with the knowledge rate (8.13%) and the demand rate (51.53%) for the service for the elderly in Lanzhou. The possible cause of the high demand rate is that Yinchuan, Karamay and Lanzhou are located in the undeveloped region of western China with a low level of economic and social development, and a high degree of an ageing population. The low knowledge rate of the service is affected by a plurality of factors, which is primarily due to the lack of publicity from the perspective of policy. The elderly aged 60 years old or above receive information via different channels from those of younger people. Effective channels that are accessible by the elderly, including television, newspapers, communities and nursing institutions should be selected for publicising the combined service, instead of new media such as the Internet, Sina blog, Weibo and WeChat etc. The publicity should also cover the elderly people’s children and family members. Government departments are responsible for strengthening the publicity in this regard. Favourable policies should be released promptly and rigorously publicised so that the elderly can obtain the favourable information quickly and effectively utilise various preferential policies provided by the country.

### Influencing factors on the demand for the combination of medical and elderly care by senior citizens in Lanzhou

#### The willingness to pay for the combination of medical and elderly care

The demand rate of the elderly willing to pay 2000 to 3000 yuan per month for purchasing the service is 49. 64%; the demand rate of seniors who are willing to pay more than 3000 yuan per month for purchasing the service is 64.50%; and 36.54% of the elderly are willing to pay no more than 1000 yuan per month for the service. According to the investigation conducted by Fan et al. on the elderly in the cities and towns of Datong, more than 50% of the elderly are willing to pay 1000 to 2000 yuan per month; 35% of the elderly are willing to pay 2000 to 3000 yuan per month; 13% of the elderly are willing to pay less than 1000 yuan per month, and only a few elderly people are willing to pay more than 3000 yuan for the service [11]. After surveying the demands of the service for the elderly in downtown Chongqing, Li et al. show that the demand rate for the service for the elderly who are willing to pay 500 to 999 yuan per month is 38.7%; the demand rate for the service by the elderly who are willing to pay 2500 to 2999 yuan per month is 8/9, and 31.0% (124/400) of the elderly are willing to pay no more than 500 yuan per month for the service [15]. The result obtained by Hu et al. surveying the elderly in Yinchuan shows that 39.59% of the respondents are willing to pay 500 to 999 yuan per month for the service; 32.99% of the respondents are willing to pay less than 500 yuan per month for the service, and only 27.41% of the respondents are willing to pay 1000 yuan or above per month for the service [16]. It is different from the result obtained in this study, which might be due to different income levels in different regions. The average monthly income of residents in Lanzhou in 2018 was about 6800 yuan [28], which was higher than that in Datong (5900 yuan), Chongqing (6400 yuan) and Yinchuan (6000 yuan) [26]. In that case, the elderly express more willingness to pay for the service in Lanzhou than in the other three cities mentioned above.

Furthermore, the demand rate for the service in Lanzhou is on the rise with the rising price that is willing to pay, which is similar to related literature [11, 15]. This is due to conditions such as social status, income and degree of education of the elderly with a high willingness to pay are better than those with a low willingness to pay [11, 15].

#### Type of medical insurance

In this study, the type of medical insurance is a factor influencing the demand rate of the service combining medical and elderly care for the elderly. The elderly with urban medical insurance have an increasing demand for the service. In general, the cost of nursing the elderly in nursing institutions is higher than home-based nursing. Also, the reimbursing proportion of urban medical insurance is high. Although many nursing charges are not included in the reimbursement category, costs incurred by the service combining medical and elderly care can be covered by urban medical insurance, which can alleviate part of the costs for the service combining medical and elderly care. In consequence, the elderly with urban medical insurance are more willing to select an institution with the elderly nursing service for their nursing needs. Some studies indicate that medical insurance is a factor influencing medical rehabilitation and health care services for the elderly [43]. Another study demonstrates that the elderly participating in the new rural cooperative medical system are more willing to have the service, in comparison to those with the medical insurance for urban residents [44]. It is probably because of different reimbursement proportions of various medical insurance systems.

#### Number of children

This study shows that the number of children is a factor influencing the demand rate of the service. The elderly with fewer children have an increasing demand for the service. This may be caused by the family miniaturisation and the increase in empty-nest families. In this way, the children cannot take care of their elderly parents. Generally speaking, the elderly with more children have richer family-supporting resources, more secure family-supporting care and lower demands for the service combining medical and elderly care. When a core family is formed, the number of elderly people requiring care is increased, while the number of family members who can take care of the elderly has decreased. In that case, the elderly cannot be cared for in an all-round way. What is worse, family members could be helpless in handling the special requirements of treatment, nursing, rehabilitation and hospice for the disabled elderly, the elderly with chronic diseases and being susceptible to disease as well as those suffering from terminal diseases. Therefore, the uncared for elderly person who has difficulty in medical treatment due to a few children or children not around has a higher demand for the service combining medical and elderly care.

#### Level of awareness of the combination of medical and elderly care

The knowledge rate of the service combining medical and elderly care in this study is one of the factors influencing the demand for the service. As can be seen from the studies conducted in Zhanjiang and Urumqi, the elderly who knew and understood the service have a higher demand rate for the service in comparison to those who had never heard of the service [31, 36]. However, the knowledge rate (8.12%) of the service is greatly inconsistent with the demand rate (51.43%) of the service. It is primarily because the service mode in Lanzhou is still under development with incomplete organisation, lack of publicity, meaning that the elderly barely know of it. Most of the elderly have never heard of or only heard of it from television and newspapers and other media without understanding or knowing little about the service. Moreover, some of the elderly cohort cannot understand the service mode, service purpose or service concept accurately and have doubts about the service model. Some even consider that the service is equivalent to the nursing service in traditional nursing centres and care homes [11]. The elderly nursing model proposed is a new elderly nursing model. The elderly with an in-depth understanding of the new model and its advantages are more willing to select the institution combining medical and elderly care for nursing. It shows that the development of the elderly nursing mode combining medical and elderly care is greatly correlated to the understanding of residents. Therefore, relevant government departments should publicise the elderly nursing mode well to enhance the knowledge and recognition of the new mode among the people.

#### Self-assessment of health

The elderly’s health self-rating status in this study is a factor in measuring the health status of the elderly. The elderly’s conscious need for elderly nursing or medical treatment can be reflected in the health self-rating status. According to the single factor analysis results, a statistically significant difference can be found in the health self-rating status in the demands of the new service (*χ*^2^ = 31.027, *P* = 0.000). The elderly with poor health self-rating status have a higher demand for the service than those with better health self-rating status. The demand rate for the service for the elderly with poor health self-rating status is 52.78%; the demand rate for the elderly with not good enough health self-rating status is 52.27%, and the demand rate for the elderly with a good health self-rating status is 41.57%. After surveying the elderly in cities and towns of Datong, Fan et al. found that the elderly with a poor health self-rating status had a higher demand for the service; the demand rate for the service by the elderly with poor health status reached 66%, while the demand rate by those with good health status was only 37%. Another study conducted in Urumqi shows that the ageing population with poor health self-rating status have a higher demand for the service than those with good health self-rating status, and the demand rate for the service for the elderly with poor health self-rating status reached 68.74%; the demand rate among the elderly with not good enough health self-rating status was 46.19%; and the demand rate by the elderly with a good health self-rating status was 53.45%. The findings in this study are consistent with those arrived at in the above studies [11, 36]. The elderly’s physical function will gradually deteriorate with an increased incidence rate of various diseases and worsening health conditions with increasing age. What is worse, some of the elderly might not be able to take care of themselves. Along with these situations, there will be an increasing demand for the service of medical and elderly care. That is the reason why the elderly with poor health status have a strong demand for the service.

#### Current elderly care model

This study indicates that the current model of elderly nursing is a factor influencing the demand for the proposed new service for the elderly. Most of the elderly consider home-based care the ideal way of delivering elderly nursing due to the influences of their physical conditions, children’s time and energy, local cultural habits, economic factors and psychological needs. Nevertheless, 42.13% of the elderly consider that there is a gap between the existing elderly nursing mode and the ideal one due to financial difficulties, unattended living, poor health and suffering from illness, no entertainment and loneliness and other reasons [16]. The mode combining medical and elderly care prioritises the elderly’s health and medical services, which differs from the traditional way of providing high-quality elderly nursing services for satisfying the elderly’s basic living needs. It is appealing to the elderly to some extent.

### Comparison between primary influencing factors on the demand for the combination of medical and elderly care in a different study

**Fig. 3:**
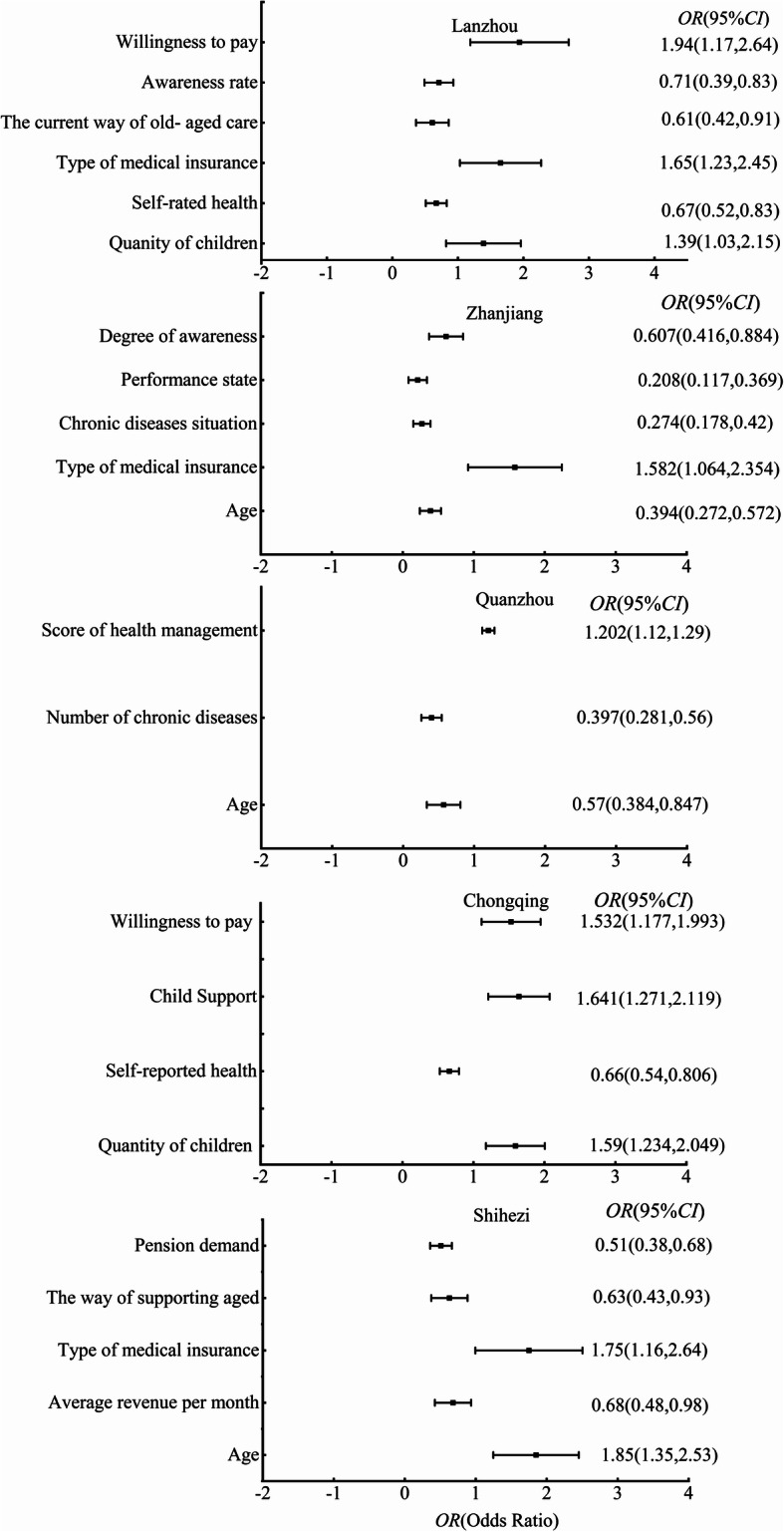
Comparison of primary factors influencing the elderly’s needs of combining medical treatment and nursing in different study

We also compared primary factors influencing the elderly’s needs for combining medical treatment and nursing in a different study. To ensure the comparability of the data, we collected data from literature reports using the same questionnaire as in this study [15, 31–33]. In the above literature, the influencing factors on the demand for the combination of medical and elderly care were analysed by binary logistic regression analysis. Therefore, we used the forest map to compare the differences between the factors affecting different studies. As can be observed from Fig. 3, the primary influencing factors of the elderly’s demand for the service are varied. The primary influencing factor of the elderly’s demand for the service is a willingness to pay in Lanzhou, children’s support in Chongqing [15], the type of medical insurance in Zhanjiang [31], health management in Quanzhou [32] and age in Shihezi [34]. The above results show that related departments should formulate and develop planning and policies for the service following the actual situation.

## Conclusions

A low level of understanding on the part of the elderly and the demand for the service combining medical and elderly care can be witnessed in Lanzhou. Meanwhile, the number of children, types of medical insurance and willingness to pay for the combination of medical and elderly care are major influencing factors among the complex factors influencing the elderly’s demand for the service proposed Lanzhou. Relevant departments in Lanzhou should improve relevant laws and regulations and release systems and standards concerning the service according to local conditions, while vigorously enhancing the publicity of the service to raise the demand for it. What is more, the construction of institutions tasked with developing the service should be accelerated to safeguard the capacity of providing the service combining medical and elderly care.

## Supplementary information

**Additional file 1.**

## Data Availability

The datasets used and/or analysed during the current study are available from the corresponding author on reasonable request.

## Abbreviations

OR: Odds ratio
PACE: Program of all-inclusive care for the elderly
95%CI: 95% Confidence interval
US: United States

## References

[1] National Bureau of Statistics (2019). Statistical bulletin of the People's Republic of China on national economic and social development 2018.

[2] Zhu Y. Interpretation of "the 13th five-year plan for the development of national undertakings for the aged and the construction of the old-age care system". http://www.gov.cn/zhengce/2017-03/15/content_5177770.html, 2017-03-15 (in Chinese).

[3] Guo ZM (2019). Research on urban "combination of medical care and nursing care" from the perspective of demand -- a case study of Taiyuan city. Master Thesis.

[4] Wu SY (2017). Research on the development status and countermeasures of the mode of combining medical care with old-age care in Chongqing. Master Thesis.

[5] Zhang YM (2016). Research on medical care and nursing service for disabled elderly in China -- based on the investigation of Qingdao city. Master Thesis.

[6] Guo C (2016). A comparative study on the combination of medicine and nursing in China and foreign countries. Master Thesis.

[7] Liu SJ (2017). Current situation analysis and countermeasure research on the mode of combining medical care with old-age care in China. Master Thesis.

[8] Liu WH, Peng JL (2015). Nursing service promotes the development of the mode of "combining medical care with nursing care". J Nurs Adm.

[9] Huang ZY (2016). Discussion on the service mode of "combining medical care with nursing care"—a case study of a nursing home for the aged in B city. J Econ Soc Dev.

[10] Liu H (2014). Thoughts and Suggestions on promoting "medical and nursing integration" in Shanghai. J Sci Dev.

[11] Lai MQ (2019). From the perspective of planning behavior theory, the study on the elderly’s willingness to "combine medical care with nursing care" in institutions in the main urban areas of Chongqing and its influencing factors. Master Thesis.

[12] Fan YF (2019). Analysis on the demand of the combination of medical care and nursing for the aged in a certain area and its influencing factors. Master Thesis.

[13] Deng DS, Wang K (2015). Comparison of foreign home-based old-age care model and its enlightenment to China. J Hebei Norm Univ (Philos Soc Sci Ed).

[14] Mui AC (2001). The program of all-inclusive care for the elderly(PACE):an innovative long –term care model in the United States. J Aging Soc Policy.

[15] Sakakibara KKK (2014). The role of public heath nurses in Japanese long-term care prevention projects in the community. J Nurs Care.

[16] Li XM, Feng ZY, Cheng QX, Wang X, Feng D (2016). Study on the demand and influencing factors of the combination of medical care and nursing for the aged in the main urban areas of Chongqing. J Gen Pract.

[17] Hu Q, Lang Y, Ma GD, Xu N, Wen HD (2019). Research on the demand of combination of medical care and nursing for the elderly in Yinchuan city and its influencing factors. J Prim Health Care.

[18] Zhou Y, Tian R, Yao H, Wang Y, Liu W (2019). Analysis on the awareness rate and influencing factors of medical and nursing care for the aged population in Urumqi. J Xinjiang Med Univ.

[19] Wang SY, Lv J, Liu X, Zhang CC (2018). Analysis on the demand and influencing factors of the combination of medical care and nursing for the aged in Changchun city. J Integr West Chin Nurs.

[20] Fang PQ (2017). Report on the development of China's medical and health services 2016 -- a special topic on the reform and development of China's medical insurance system.

[21] Zhao M (2009). Review and prospect of China's medical insurance system reform. J Hubei Soc Sci.

[22] Ministry of Human Resources and Social Security of the People's Republic of China (2016). China's social insurance development annual report 2015.

[23] Wang PQ, Lei YR, Lv PS (2018). An analysis on the construction of the combination mechanism of medical and nursing care beyond multiple games -- the dilemma and the way out of the model of the combination of medical and nursing care in China. J Chin Acad Governance.

[24] Zhou XL, Jiao YH, Wang C (2019). Problems and countermeasures of medical and nursing service system in xi 'an old-age care institutions. Chin J Gerontol.

[25] Ma LL, Chen N, Tang SL (2016). Research on the development policy of medical and nursing combined with pension service in pension institutions. J Med Soc.

[26] Ni YC, Wang CQ, Chen N (2016). Research on the model of the combination of medical and nursing institutions in China under the background of aging. J Med Soc.

[27] National Bureau of Statistics (2019). China statistical yearbook.

[28] Gansu Statistics Bureau (2019). Gansu statistical yearbook.

[29] Lanzhou Statistics Bureau (2019). Lanzhou statistical yearbook.

[30] Li YM, Wei YQ, Pei ZX (2018). Analysis on the development status and countermeasures of "combination of medicine and nursing" in Lanzhou city. J Prim Health Care.

[31] Gao CY (2019). Research on the development status, problems and countermeasures of the combined medical and nursing mode in Lanzhou city. Master Thesis.

[32] Wu LJ, Liao SL, Wen RL, Ni SK (2019). Analysis on the demand and influencing factors of the combination of medical care and nursing for the aged in urban area of Zhanjiang city. J Nurs Res.

[33] Huang SF, Huang FF, Gong GM, Chen LX (2017). A study on the demand and influencing factors of community empty-nest elderly people in Quanzhou city for combined medical and nursing services. J Nurs Res.

[34] Wu Y, Wang XN, Yu Q, Ma GF (2019). Recognition of medical care and pension mode for the elderly in Shihezi city. Chin J Gerontol.

[35] Dong Q (2019). Research on the mode of medical care combined with old-age care service in Gansu province. Master Thesis.

[36] Chen JY (2019). Research on the mode of combination of medical care and nursing and the health care of the elderly under the background of healthy aging. Doctor Thesis.

[37] Tian R (2019). Medical and nursing care for the aged population in Urumqi combined with cognitive status and demand analysis. Master Thesis.

[38] Liu W, Zhou Y, Ge L, Wang Y (2019). Analysis of the current situation of medical and nursing needs of the elderly population in Karamay and its influencing factors. J Xinjiang Med Univ.

[39] Wang Y, Xu Y, Wang M, Wei JM (2019). Investigation and research on the cognition status of community traditional Chinese medicine medical and nursing mode for elderly residents in Beijing. Chin J Med.

[40] Li T, Chen JL (2015). Analysis on the cognition, attitude and influencing factors of Tianjin residents on the combination of medical care and nursing. J Tech Innov.

[41] Ji KG, Shi X, Guan CM (2018). Analysis on the demand and influencing factors of combined medical and nursing services for middle-aged and elderly patients in Weifang city. Chin J Geriatric Care.

[42] Zhang XF, Wang XF, Jin Y, Jiang XB (2017). Analysis on the characteristics and influencing factors of the elderly’s demand for combination of medical care and nursing. J Prim Health Care.

[43] Wu K, Cao PY, Qian JH (2017). Analysis on the cognition status and influencing factors of "combination of medical care and nursing care" mode for the elderly in Chengdu. J Sichuan Univ (Med ED).

[44] Huang FY, Chen F, Tao HY, Li XR (2017). Factors influencing the service demand of "combination of medical care and nursing care" for the elderly. Chin J Gerontol.

