# Supplementary material for: Analysis of the status quo of the Elderly’s demands of medical and elderly care combination in the underdeveloped regions of Western China and its influencing factors: a case study of Lanzhou
Source: BMC Geriatr. 2020 Sep 9;20:338. doi: 10.1186/s12877-020-01616-6 (PMC7488146; doi:10.1186/s12877-020-01616-6)
Supplement: Supplementary file 1 — Additional file 1. [file 12877_2020_1616_MOESM1_ESM.docx]

A Survey on the Demand for the Combined Medical and Elderly Care Services in Lanzhou and Its Influencing Factors

To whom it may concern:

Hello! This survey is intended to help us understand the awareness and demand of the elderly on the combined medical and elderly care services and its influencing factors. The contents of all questionnaires will be kept completely confidential and only used by researchers for data analysis. All you need to do is to fill in the questionnaire based on your actual situation. Your answer will provide a valuable reference for us to draw correct conclusions. We really appreciate your kind support and contribution. Many thanks!

I. Basic information

A1. Your gender is ()

1. Male 2. Female

A2. Your age is ()

1. 60-70 years 2. 71-80 years 3. >80 years

A3. Your marital status is ()

1. Married 2. Unmarried 3. Divorced or widowed

A4. The highest educational attainment you completed is ()

1. Elementary school and below 2. Junior high school 3. Senior high school or technical secondary school 4. Junior college 5. Bachelor degree or above

A5. The occupation before you retired is ()

1. State functionary 2. Public institution personnel 3. Enterprise staffs 4. Others

A6. The number of children you have raised is ()

1. Zero 2. One 3. Two 4. Three or above

II. Health condition

B1. Your physical condition is ()

1. Good 2. Fair 3. Poor

B2. Do you suffer from any chronic diseases?

1. Yes 2. No

B3. Which option can best describe your self-care and daily living skills?

1. I can take care of myself 2. I need help from others 3. I can't take care of myself at all

III. Healthcare, insurance and pension

C1. The type of your medical insurance is ()

1. The medical insurance for urban workers 2. The medical insurance for urban and rural residents 3. Commercial health insurance 4. None

C2. The type of your endowment insurance is ()

1. The endowment insurance for urban workers 2. The endowment insurance for urban and rural residents 3. Enterprise annuity 4. Commercial endowment insurance 5. None

C3. Your monthly income is () RMB per month

1. <2000 2.2000-4000 3.4000-6000 4. >6000

C4. Your living mode is:

1. Live with others 2. Live alone

C5. The way of your old-age care is:

1. Family endowment 2. Community endowment 3. Institution endowment 4. Others

IV. The awareness and demand on the combined medical and elderly care services

D1. Have you ever heard of the combined medical and elderly care services?

1. Never heard 2. Heard but didn’t know the details 3. Understand some details 4. Understand very well

D2. Do you have the need to get the combined medical and elderly care services?

1. Yes 2. No

D3. What are your requirements for the elderly care?

1. Medical care 2. Assisted living 3. Mental care 4. Leisure and entertainment

D4. If you want to get the combined medical and elderly care services, the average expense you can afford is () RMB/month

1. <1000 2.1000-2000 3.2000-3000 4. >3000

This is the end of your questionnaire. Thank you for your active participation and inputs. Wish you a happy life!
